# Impact of abdominal aortic aneurysm screening on quality of life

**DOI:** 10.1002/bjs.10721

**Published:** 2018-02-05

**Authors:** M F Bath, D Sidloff, A Saratzis, M J Bown, R Pathak, M Brooks, P Hayes, C Imray, J Quarmby, S Choksy, J J Earnshaw, C P Shearman, E Grocott, T Rix, I Chetter, W Tennant, G Libertiny, T Sykes, M Dayer, L Pike, A Pherwani, C Nice, N Browning, C McCollum, S Yusuf, M Gannon, J Barwell, S Baker, S R Vallabhaneni, A Davies

**Affiliations:** Department of Cardiovascular Sciences and National Institute for Health Research Leicester Biomedical Research Unit, University of Leicester, Leicester Royal Infirmary, Leicester, UK

## Abstract

**Background:**

Screening for abdominal aortic aneurysm (AAA) is known to reduce AAA-related mortality; however, the psychological impact of population AAA screening is unclear. The aim was to assess the impact of AAA diagnosis on quality of life (QoL) using data from an established AAA screening programme.

**Methods:**

Mental and physical QoL scores for men diagnosed with AAA through participation in the English and Welsh AAA screening programmes were compared with no-AAA controls. Participants were identified through the United Kingdom Aneurysm Growth Study (UKAGS), a nationwide prospective cohort study of men with an AAA of less than 55 mm diagnosed through voluntary participation in screening. The UKAGS participants completed QoL questionnaires at the time of screening and annually thereafter.

**Results:**

A transient reduction in mental QoL scores was observed following the diagnosis of AAA, returning to baseline levels after 12 months. Physical QoL remained consistently lower in the AAA cohort. Participants thought about their AAA and the AAA growth progressively less 12 months after the initial screening diagnosis. AAA growth rate had no influence over QoL parameters.

**Discussion:**

This study suggests that screening for AAA does reduce mental QoL; however, this effect is transient (less than 12 months). Men diagnosed with AAA have a consistently worse physical QoL compared with controls.

## Teaser

Small initial reduction

## Introduction

Abdominal aortic aneurysm (AAA) is defined as an abnormal dilatation of the abdominal aorta of 30 mm or more, and constitutes a significant health problem worldwide^1^. Each year in England and Wales, AAAs cause over 4000 deaths following aortic rupture^2^, with approximately 8000 patients a year undergoing surgery to prevent this^3^. In 2013, the National Health Service (NHS) AAA screening programme (NAAASP) was fully rolled out across England, based on evidence from several RCTs suggesting that AAA-related mortality was reduced through participation in AAA screening^4^. The NAAASP currently invites all men in their 65th year to receive a one-off non-invasive abdominal ultrasound scan. In England in 2015–2016, 227 543 men were screened and 2549 (1·1 per cent) were diagnosed with an AAA; however, only 723 men (0·3 per cent) had an AAA large enough (at least 55 mm) to require referral for consideration of surgery^5^. This highlights one of the major issues with screening for AAA in that, although it remains cost-effective, the majority of patients identified do not require immediate surgery and are subsequently entered into ongoing surveillance, either 6-monthly or annually. Most men with a screen-detected AAA will spend 3–5 years in surveillance before reaching the threshold for elective AAA repair, rising to over 7 years for men with a 30-mm AAA^6^. Currently 13 104 men in England are in AAA surveillance^5^.

This has led to questions being raised over the psychological impact of AAA screening. Some have even suggested that AAA screening may do more harm than good^7^. A small number of observational studies have investigated quality of life (QoL) in those who are identified at screening to have an AAA^8–13^, demonstrating varying results and conclusions when comparing screened and unscreened cohorts.

The United Kingdom Aneurysm Growth Study (UKAGS) is a prospective observational cohort study currently recruiting men with AAA identified through the English NHS AAA screening programme (NAAASP) and the Welsh AAA screening programme (WAAASP), with the aim of investigating the growth rates of small AAAs. All recruited men (those with an AAA and controls without) are sent an annual self-completed postal questionnaire to obtain longitudinal clinical and QoL data. This resource was used to assess the contemporary impact of screening for AAA on men who attend the NAAASP and WAAASP.

## Methods

NAAASP and WAAASP invite all men in England and Wales during their 65th year of age to attend AAA screening^14^. Eligible men are sent an invitation letter to attend a local clinic for an ultrasound scan. A technician measures the maximal anteroposterior inner wall to inner wall diameter of the infrarenal aorta. Those with a diameter of less than 3 cm are discharged; those with a diameter between 3·0 and 5·4 cm are offered ultrasound surveillance every 6 or 12 months (based on AAA diameter); and men with an aortic diameter above 5·4 cm are directly referred for possible surgical repair.

UKAGS is a prospective observational cohort study that recruits men with an AAA, as well as individuals without AAA (controls) who have attended AAA screening, from NAAASP or WAAASP^15^. All recruited men are sent annual self-completion postal questionnaires (*Appendix S1*, supporting information) to obtain longitudinal clinical information and QoL information. Additionally, those with AAA on initial screening undergo annual ultrasound screening measurements through the standard surveillance procedures^14^. UKAGS is currently recruiting individuals from 14 units across England and Wales, and aims to recruit 20 000 men over 5 years. Ethical approval has been granted by an NHS research ethics committee, and men have provided their written informed consent upon recruitment for QoL data collection and analyses.

### Data collection and quality-of-life assessments

Data collected at baseline included AAA diameter (inner wall to inner wall measurement), demographics, standard cardiovascular co-morbidities and QoL-related fields. This includes eight questions adapted from the Medical Outcomes Study Short Form 36 questionnaire (SF-8), a survey that has previously been recommended specifically for vascular disease-related QoL outcome analyses^16^ and has demonstrated high reliability and validity^17^. Several other QoL questionnaires are available, and have been used by other groups. The present questions were considered most suitable for the UKAGS.

The SF-8 questionnaire uses single-item scales addressing eight domains of general health, physical functioning, role limitations (due to physical health), bodily pain, vitality, social functioning, mental health and role limitations (due to emotional health). These parameters are then used to produce two outcome measures of QoL: the Physical Component Summary (PCS) and the Mental Component Summary (MCS). The scores range from 0 to 100, where zero indicates the lowest and 100 the highest level of health, calibrated so that the average score is 50, with a standard deviation of 10^17^. Scores were calculated using the QualityMetric Health Outcomes™ Scoring Software 4.5 (Optum®; QualityMetric, Lincoln, Rhode Island, USA). Additionally, using a Likert scale (1, not at all; 5, all the time), men with a known AAA were asked how often they thought about their aneurysm and how often they had thought about the potential for aneurysm growth in the preceding 4 weeks.

As recruitment occurred through the traditional screening pathway, participants were recruited into the study across an extended period (September 2011 to July 2015). Relative to each man's baseline recruitment date, QoL data collection follow-up was divided into four groups after initial screening: 0–12, 13–24, 25–36 and 37 or more months. The primary outcome measure was comparing PCS and MCS at each time point between men with a diagnosed AAA on initial screening and those without an AAA (control group).

Data collection was done at yearly follow-up intervals with recruitment from NAAASP and WAAASP into the study occurring continuously throughout the 4-year study. Thus, participants were in the study for varying lengths of time and had completed a varying number of questionnaires (*Table 1*).

**Table 1:** Total number of questionnaire respondents for each time interval after initial screening

Time from initial screening (months)	No aneurysm	Aneurysm	Total
0–12	4807	174	4981
13–24	4232	238	4470
25–36	914	142	1056
≥ 37	52	93	145

### Statistical analysis

The SF-8 QoL data collected were analysed using ANOVA, comparing the AAA group with the no-AAA group at each interval. Regression analyses accounted for potential confounding factors, including demographics and co-morbidities. Mean growth rate (cm/month) during surveillance was calculated and linear regression was used to identify any association of growth rate with the QoL parameters. ANOVA was used to assess changes in the frequencies over time with which respondents thought about their aneurysm and aneurysm growth; data for the intervals of 13–24, 25–36 and 37 or more months were compared with those for the 0–12-month interval, which acted as a baseline value. Continuous variables are presented as mean(s.d.) or mean(s.e.m.) values, as appropriate. A Pearson χ^2^ test was used to compare categorical variables and a paired *t* test to compare continuous data. Data were analysed using IBM SPSS® version 22.0 (IBM, Armonk, New York, USA). *P* < 0·050 was considered statistically significant.

## Results

A total of 5011 men were recruited into the study, of whom 381 (7·6 per cent) had an AAA identified via screening. Overall, they were followed for a mean of 19·0(9·1) months from their initial screening appointment.

Men with an AAA were older (age 72·6 *versus* 69·8 years; *P* < 0·001), had a higher BMI (28·1 *versus* 27·0 kg/m^2^; *P* < 0·001) and were more likely to be a current smoker (15·1 *versus* 5·2 per cent; *P* < 0·001) than those in the control group (*Table 2*). When comparing co-morbidities to the control group, men with an AAA were more likely to have diabetes mellitus (18·8 *versus* 10·0 per cent), ischaemic heart disease (12·2 *versus* 4·4 per cent), high cholesterol (53·2 *versus* 30·8 per cent), previous stroke (6·1 *versus* 2·9 per cent) and a previous myocardial infarction (21·1 *versus* 5·8 per cent) (all *P* < 0·001).

**Table 2:** Demographics of men included

	No AAA (n = 4630)	AAA (n = 381)	*P* ^†^
Age (years)*	69·8(3·5)	72·6(5·5)	< 0·001^‡^
BMI (kg/m^2^)^*^	27·0(4·4)	28·1(4·3)	< 0·001^‡^
AAA diameter at initial screening (mm)*	17·8(0·2)	36·1(0·7)	< 0·001^‡^
Current smoker	237 of 4596 (5·2)	57 of 378 (15·1)	< 0·001
Diabetes mellitus	454 of 4536 (10·0)	69 of 367 (18·8)	< 0·001
IHD	197 of 4507 (4·4)	43 of 352 (12·2)	< 0·001
High cholesterol	1401 of 4542 (30·8)	194 of 365 (53·2)	< 0·001
Previous stroke	134 of 4612 (2·9)	23 of 376 (6·1)	< 0·001
Previous MI	269 of 4614 (5·8)	80 of 379 (21·1)	< 0·001

Values in parentheses are percentages unless indicated otherwise;

* values are mean(s.d.). AAA, abdominal aortic aneurysm; IHD, ischaemic heart disease; MI, myocardial infarction.

† Pearson χ^2^ test, except

‡ paired *t* test.

### Quality of life

**Fig. 1:**
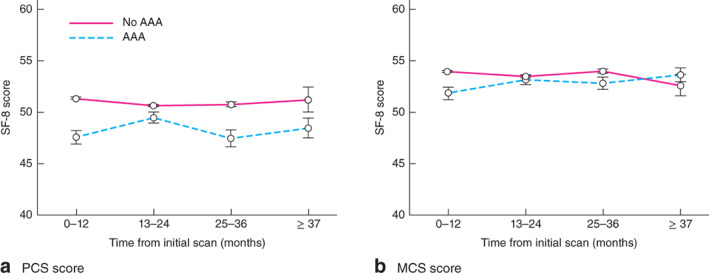
Changes in SF-8 scores for **a** Physical Component Summary (PCS) score and **b** Mental Component Summary (MCS) score after initial screening. Values are mean(s.e.m.). The SF-8 questionnaire included eight questions adapted from the Medical Outcomes Study Short Form 36

For the PCS, scores in the AAA group were significantly lower at 0–12, 13–24 and 25–36 months than those in the control group (*P* < 0·001, *P* = 0·028 and *P* < 0·001 respectively) (*Table 3* and *Fig. 1a*). Over 37 months after screening, differences in PCS were non-significant. For the MCS, scores were significantly lower immediately after screening in men with an AAA *versus* the control group (*P* < 0·001) (*Table 4* and *Fig. 1b*). However, after 12 months, MCS scores from the AAA cohort returned to baseline levels, equivalent to those of men with no AAA, and continued thus for the remainder of the follow-up.

**Table 3:** SF-8 Physical Component Summary scores for men with and those without an abdominal aortic aneurysm for each time interval after initial screening

Time from initial screening (months)	PCS score		*P* ^*^
	No AAA	AAA	
0–12	51·4(7·9)	47·6(8·9)	< 0·001
13–24	50·7(8·4)	49·5(8·7)	0·028
25–36	50·8(8·3)	47·5(10·0)	< 0·001
≥ 37	51·3(8·8)	48·5(9·4)	0·077

The SF-8 questionnaire included eight questions adapted from the Medical Outcomes Study Short Form 36. Values are mean(s.d.). PCS, Physical Component Summary; AAA, abdominal aortic aneurysm.

* ANOVA.

**Table 4:** SF-8 Mental Component Summary scores for men with and those without an abdominal aortic aneurysm for each time interval after initial screening

Time from initial screening (months)	MCS score		*P* ^*^
	No AAA	AAA	
0–12	54·0(7·0)	51·9(8·3)	< 0·001
13–24	53·5(7·5)	53·2(7·4)	0·610
25–36	54·0(7·1)	52·9(7·1)	0·884
≥ 37	52·7(7·6)	53·7(6·7)	0·408

The SF-8 questionnaire included eight questions adapted from the Medical Outcomes Study Short Form 36. Values are mean(s.d.). MCS, Mental Component Summary; AAA, abdominal aortic aneurysm.

* ANOVA.

Regression analysis was done, adjusting for the additional co-variables collected, for both PCS and MCS (*Tables S1* and *S2*, supporting information). The lower PCS scores remained significant across all AAA groups after screening (*P* < 0·001), whereas the MCS scores overall showed no differences between the AAA and control group (*P* = 0·443).

The effect of growth rate on QoL was analysed by comparing QoL to the mean growth rate (cm/month) recorded for all patients with AAA. QoL for both MCS and PCS showed no relationship with growth rate (*Figs S1* and *S2*, supporting information).

### Impact of AAA and AAA growth

**Fig. 2:**
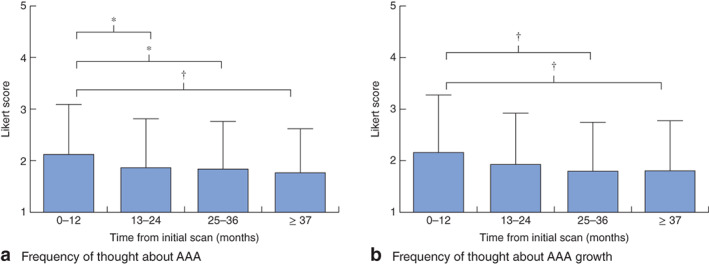
Likert scores of the frequency with which men had thought about **a** their abdominal aortic aneurysm (AAA) and **b** AAA growth in the preceding 4 weeks. Values are mean(s.d.). *P < 0·050, †*P* ≤ 0·010 (ANOVA)

ANOVA demonstrated a progressive reduction in the frequency with which men with an AAA had thought about their aneurysm in the preceding 4 weeks, at 13–24 months (*P* = 0·025), 25–36 months (*P* = 0·040) and 37 months or more (*P* = 0·005), all showing a significant reduction relative to baseline values at 0–12 months (*Fig. 2a*; *Table S3*, supporting information). When men with a small AAA were asked how often they had thought about aneurysm growth in the preceding 4 weeks, there was also a significant reduction in frequency at 25–36 months (*P* = 0·004) and 37 months or more (*P* = 0·006) (*Fig. 2b*; *Table S4*, supporting information).

## Discussion

This analysis demonstrates that men diagnosed with an AAA through screening have a transient reduction in mental QoL during the first year, but this then returns to normal. Furthermore, with time, a man diagnosed with an AAA is likely to think progressively less about it and its growth. This study also demonstrates that men with an AAA have a consistently lower physical QoL than men without an AAA, even when adjusted for co-variables.

This is not the first time that a transient impact of mental QoL has been demonstrated in screening programmes: colorectal, prostate and breast cancer programmes have all been shown to have small effects on QoL that tend to diminish with long-term follow-up^18–20^. Faecal occult blood testing for colorectal cancer had no impact on mental QoL^18^, prostate cancer screening showed no influence on physical, psychological or social functioning^19^, and in women recalled after breast cancer screening, the initial anxiety and depression associated with the appointment had decreased significantly, even after a few days^20^. However, contemporaneous data on QoL in men screened for AAA were lacking.

For AAA screening, the majority of the QoL evidence on this topic has come from the Multi-Aneurysm Screening Study (MASS)^9^. Like the present study, the longer-term mental impact appeared to be negligible in MASS. Using similar methodology, the MASS data set demonstrated a significant reduction in mental component scores for QoL 6 weeks after initial AAA diagnosis, yet, as in the present study, an improvement was seen 12 months after diagnosis, back to near-baseline values.

Smaller historical studies have shown similar findings. Lucarotti and colleagues^11^ showed that initial screening investigations caused mild anxiety that did not persist following AAA diagnosis. Wanhainen and co-workers^8^ found that only individuals with a low QoL score before screening were susceptible to potential negative effects. If such transient negative effects on mental QoL are seen in men after screening, more work is warranted to evaluate the potential benefit that could occur from introducing counselling, with discussion surrounding the presence and growth of the AAA, and how this may impact on the man with the disease.

The finding that PCS scores were lower in men with an AAA compared with controls, even after adjustment for confounders, probably reflects the co-morbid nature of many men with AAA. This observation was also seen in MASS^9^, where men with an AAA had lower physical QoL scores, from both the Short Form 36 (QualityMetric, Lincoln, Rhode Island, USA) and EuroQol – 5D (EuroQol Group, Rotterdam, The Netherlands) questionnaires. AAA is an independent marker of cardiovascular risk, with a documented 3·0 per cent per year risk of cardiovascular death in patients with a small AAA^21^. AAA and cardiovascular disease have been shown to share risk factors^22,23^.

It might be assumed that AAA growth would have a negative effect on the MCS score. Here, no association between these parameters was seen. Indeed, the longer a man had been involved in the surveillance programme, the less he reported thinking about AAA growth. These findings all support the suggestion that AAA screening does not have a significant or long-term effect on QoL. These findings were echoed by Dahlberg and colleagues^24^, who demonstrated that the reassurance a patient received from increased AAA surveillance and the positive reinforcement of screening programmes eventually outweighed the potential negative effects that might be anticipated about worsening health.

One of the key limitations of this work is the fact that QoL scores were not available before screening. As the scores of the no-AAA participants acted as a control group, and remained stable throughout the study, it can be assumed that QoL would be similar before screening. There was a discrepancy in the longer-term follow-up rate between men with an AAA and no-AAA controls, where reduced compliance may reflect decreasing engagement in those without an AAA.

The recruitment method employed meant that some men were recruited at the time of the first screening scan and some during AAA surveillance. To allow for this, the time of recruitment was recorded and used to adjust analyses of AAA growth accordingly. This is reflected in the difference in the mean age of men with and those without an AAA at baseline. It was not possible to conduct a direct regression analysis of the SF-8 data set owing to the clustered annual follow-up of the men, yet ANOVA was able to provide a more suitable and relevant alternative.

The conclusions drawn from this work are applicable only to men screened as positive for AAA. The overall effects of screening on the larger number of men with negative scans are not yet determined.

## Collaborators

Other UK Aneurysm Growth Study investigators include: R. Pathak, M. Brooks, P. Hayes, C. Imray, J. Quarmby, S. Choksy, J. J. Earnshaw, C. P. Shearman, E. Grocott, T. Rix, I. Chetter, W. Tennant, G. Libertiny, T. Sykes, M. Dayer, L. Pike, A. Pherwani, C. Nice, N. Browning, C. McCollum, S. Yusuf, M. Gannon, J. Barwell, S. Baker, S. R. Vallabhaneni, A. Davies.

## Supplementary Material

bjs10721-sup-0001-AppendixS1 **Appendix S1.** Questionnaire sent to all recruited men for self-completion
**Table S1** Regression analysis for Physical Component Summary score
**Table S2** Regression analysis for Mental Component Summary score
**Table S3** Likert scores of the frequency with which men had thought about their AAA in the preceding 4 weeks
**Table S4** Likert scores of the frequency with which men had thought about their AAA growth in the preceding 4 weeks
**Fig. S1** Linear regression for aneurysm growth rate and Physical Component Summary score
**Fig. S2** Linear regression for aneurysm growth rate and Mental Component Summary score
