# Supplementary material for: Impact of abdominal aortic aneurysm screening on quality of life
Source: Br J Surg. 2018 Feb 5;105(3):203–8. doi: 10.1002/bjs.10721 (PMC5817237; doi:10.1002/bjs.10721)
Supplement: bjs10721-sup-0001-AppendixS1 — Appendix S1. Questionnaire sent to all recruited men for self-completion Table S1 Regression analysis for Physical Component Summary score Table S2 Regression analysis for Mental Component Summary score Table S3 Likert scores of the frequency with which men had thought about their AAA in the preceding 4 weeks Table S4 Likert scores of the frequency with which men had thought about their AAA growth in the preceding 4 weeks Fig. S1 Linear regression for aneurysm growth rate and Physical Component Summary score Fig. S2 Linear regression for aneurysm growth rate and Mental Component Summary score [file bjs10721-sup-0001-appendixs1.docx]

**BJS_10721**

**Impact of abdominal aortic aneurysm screening on quality of life**

**M. F. Bath, D. Sidloff, A. Saratzis and M. J. Bown**

**Appendix S1** Questionnaire sent to all recruited men for self-completion


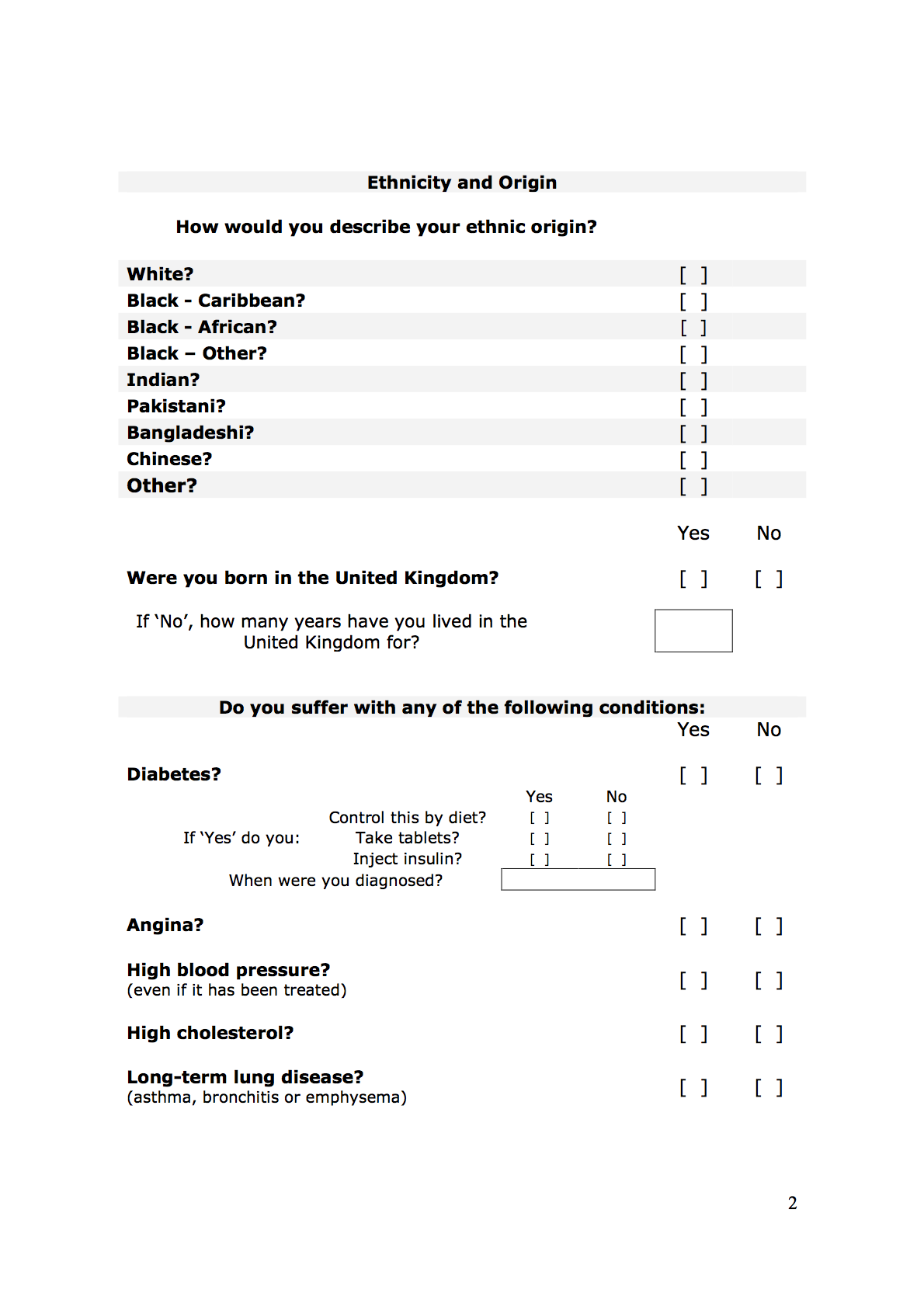


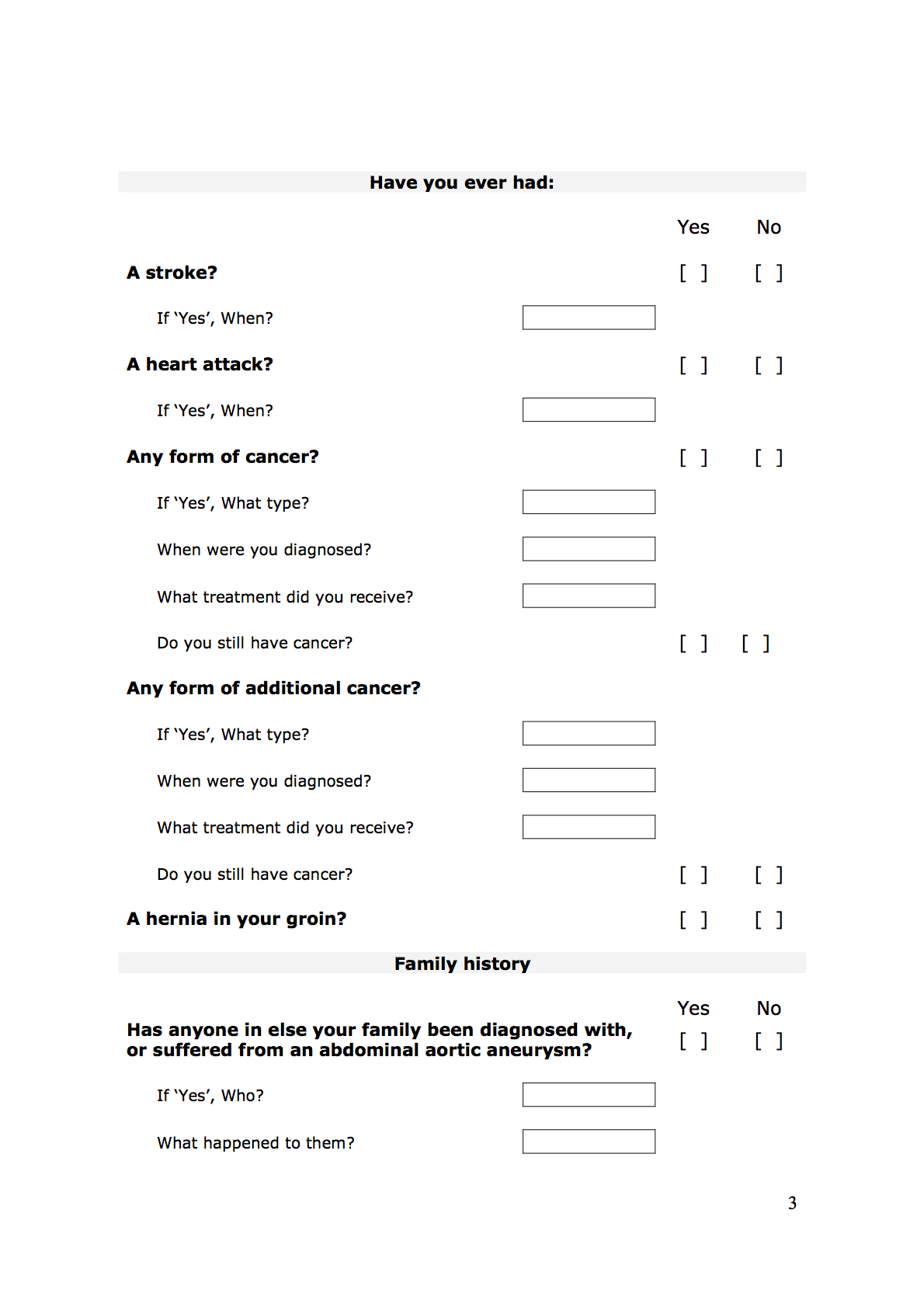


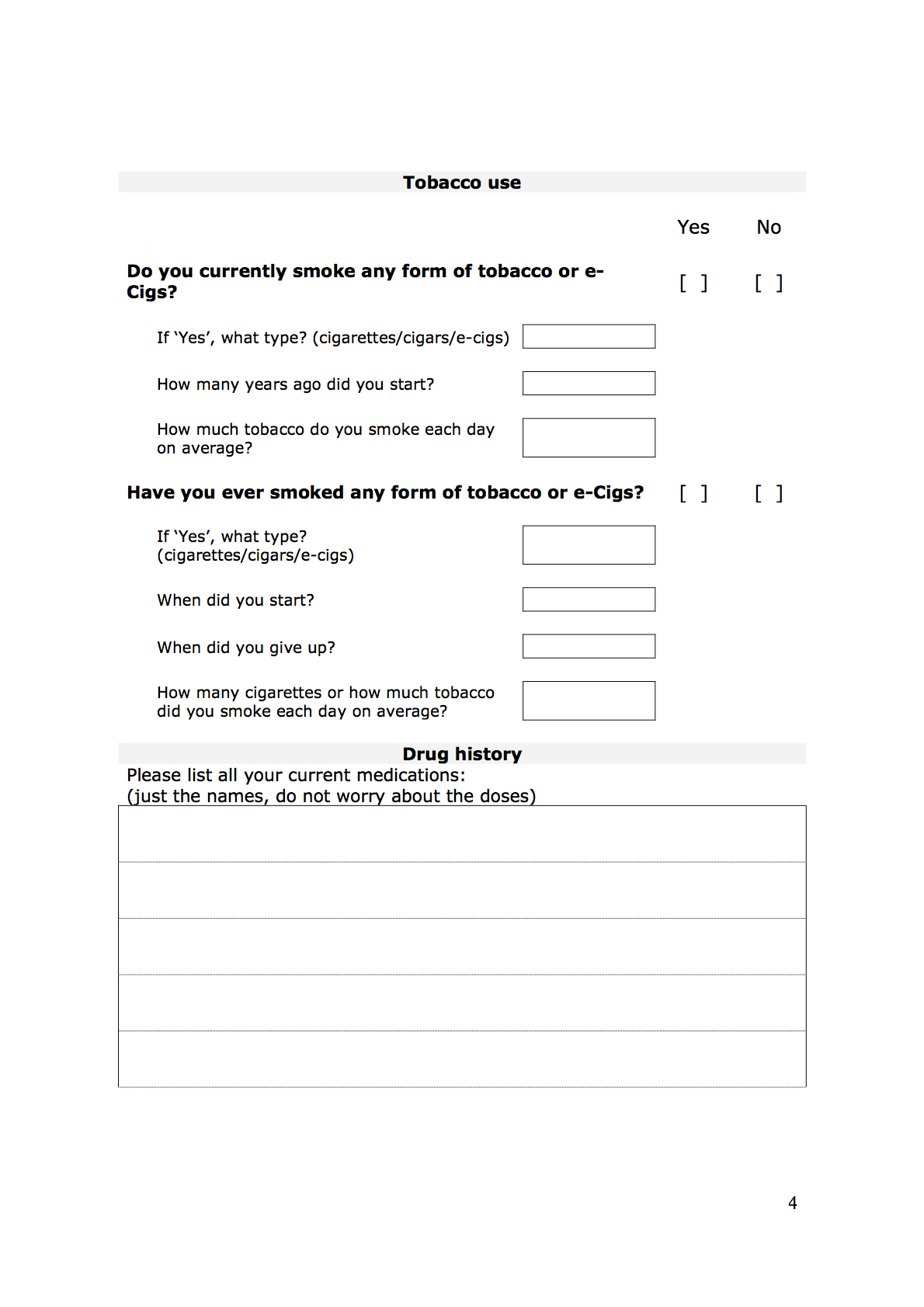


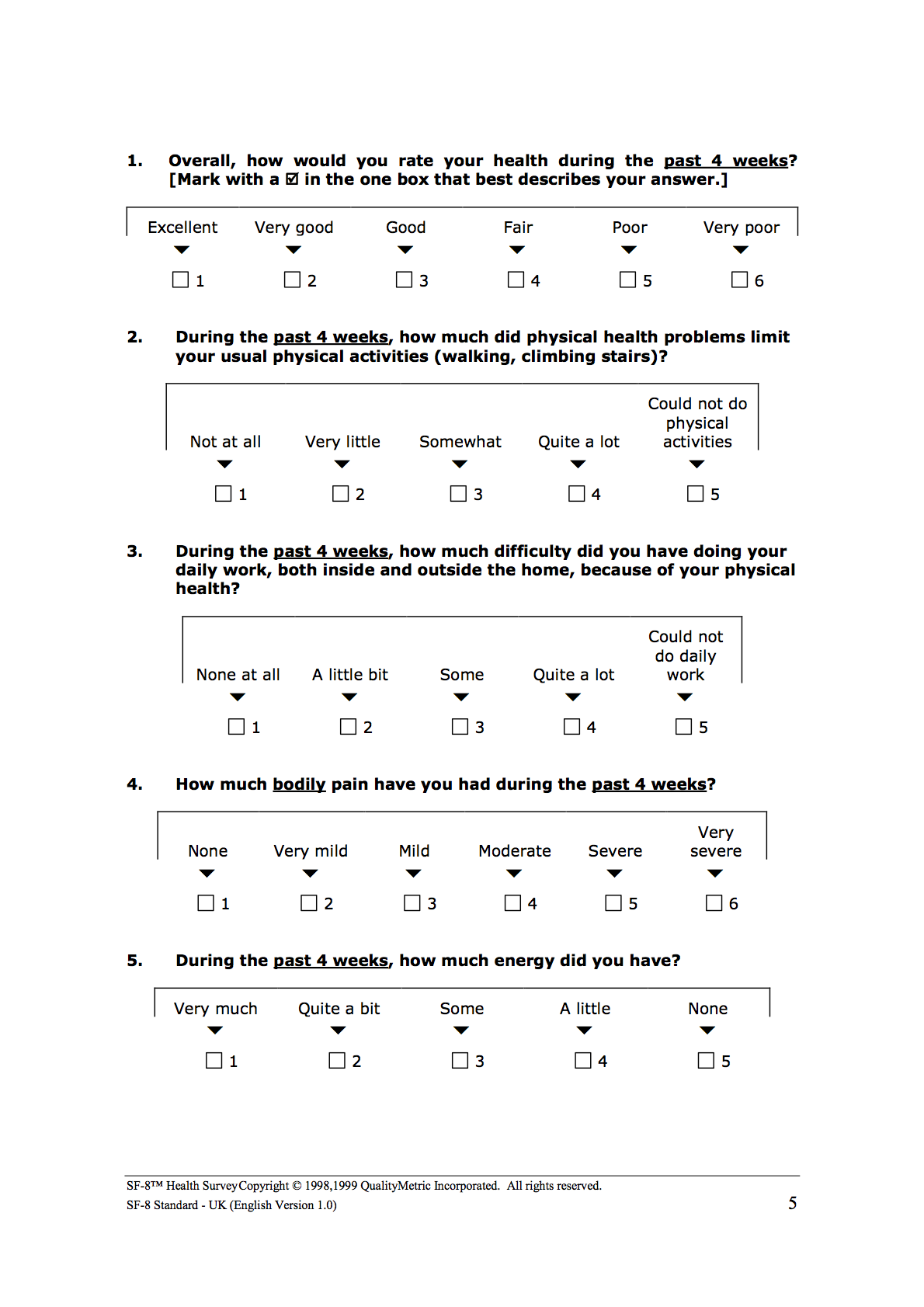


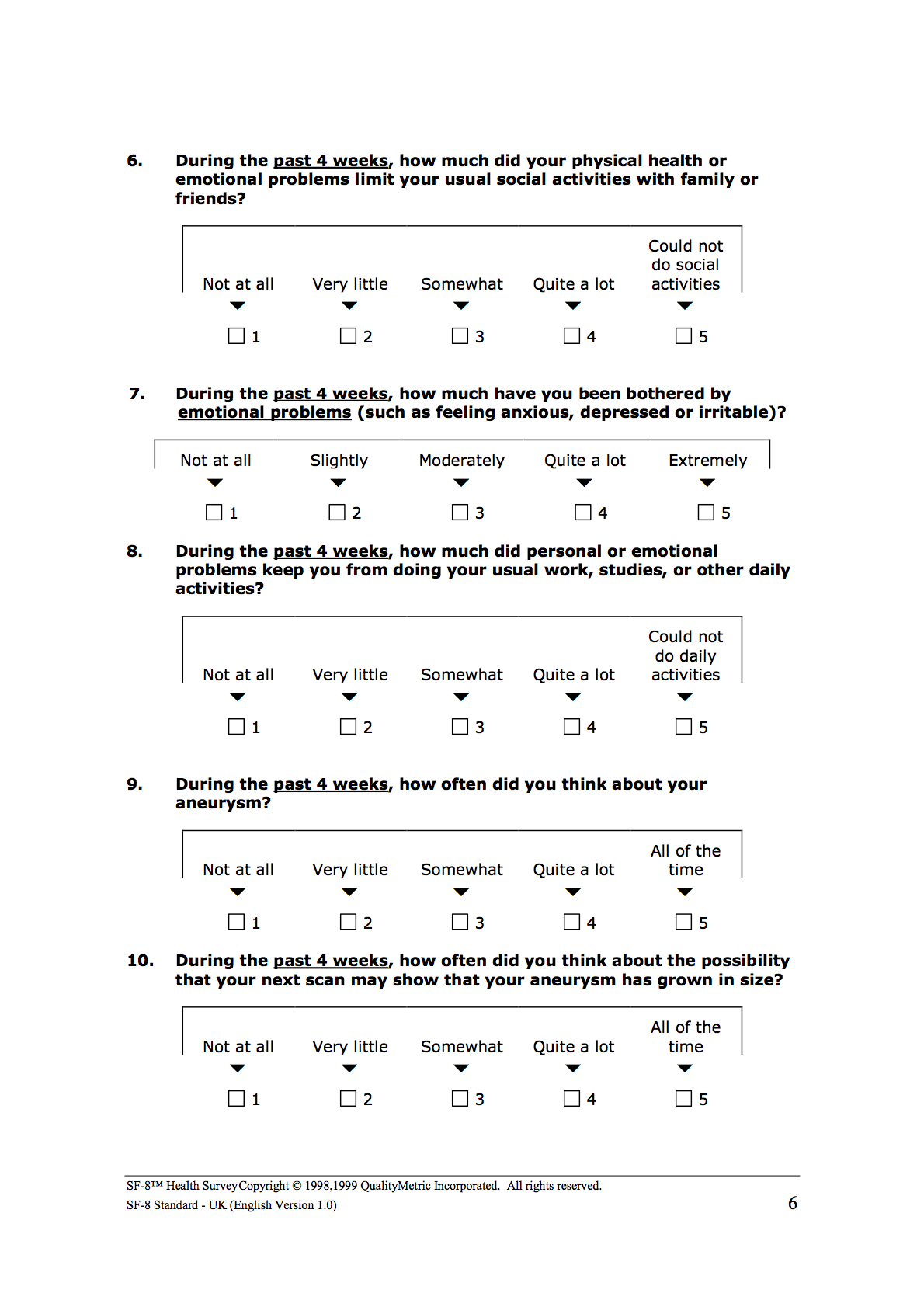


**Table S1** Regression analysis for Physical Component Summary score

| **Model Summary** | | | | | | | | | |
| --- | --- | --- | --- | --- | --- | --- | --- | --- | --- |
| Model | R | R Square | Adjusted R Square | Std. Error of the Estimate | Change Statistics | | | | |
|  |  |  |  |  | R Square Change | F Change | df1 | df2 | Sig. F Change |
| 1 | .130^a^ | .017 | .016 | 8.144 | .017 | 24.520 | 7 | 9956 | .000 |
| a. Predictors: (Constant), cancer, months, diabetes, stroke, MI, AAA, angina | | | | | | | | | |

| **Coefficients^a^** | | | | | | | | |
| --- | --- | --- | --- | --- | --- | --- | --- | --- |
| Model | | Unstandardized Coefficients | | Standardized Coefficients | t | Sig. | 95.0% Confidence Interval for B | |
|  |  | B | Std. Error | Beta |  |  | Lower Bound | Upper Bound |
| 1 | (Constant) | 51.863 | .133 |  | 389.561 | .000 | 51.602 | 52.124 |
|  | months | -.031 | .008 | -.038 | -3.739 | .000 | -.047 | -.015 |
|  | AAA | -2.067 | .358 | -.059 | -5.775 | .000 | -2.768 | -1.365 |
|  | diabetes | -1.473 | .282 | -.052 | -5.224 | .000 | -2.026 | -.921 |
|  | angina | -2.259 | .428 | -.055 | -5.278 | .000 | -3.098 | -1.420 |
|  | stroke | -1.455 | .490 | -.030 | -2.971 | .003 | -2.416 | -.495 |
|  | MI | -.804 | .364 | -.023 | -2.212 | .027 | -1.517 | -.092 |
|  | cancer | -.490 | .246 | -.020 | -1.992 | .046 | -.972 | -.008 |
| a. Dependent Variable: PCS | | | | | | | | |

**Table S2** Regression analysis for Mental Component Summary score

| **Model Summary** | | | | | | | | | |
| --- | --- | --- | --- | --- | --- | --- | --- | --- | --- |
| Model | R | R Square | Adjusted R Square | Std. Error of the Estimate | Change Statistics | | | | |
|  |  |  |  |  | R Square Change | F Change | df1 | df2 | Sig. F Change |
| 1 | .080^a^ | .006 | .006 | 7.160 | .006 | 9.109 | 7 | 9949 | .000 |
| a. Predictors: (Constant), cancer, months, diabetes, stroke, MI, AAA, angina | | | | | | | | | |

| **Coefficients^a^** | | | | | | | | |
| --- | --- | --- | --- | --- | --- | --- | --- | --- |
| Model | | Unstandardized Coefficients | | Standardized Coefficients | t | Sig. | 95.0% Confidence Interval for B | |
|  |  | B | Std. Error | Beta |  |  | Lower Bound | Upper Bound |
| 1 | (Constant) | 54.142 | .117 |  | 462.350 | .000 | 53.913 | 54.372 |
|  | months | -.012 | .007 | -.017 | -1.701 | .089 | -.027 | .002 |
|  | AAA | -.241 | .315 | -.008 | -.767 | .443 | -.859 | .376 |
|  | diabetes | -.417 | .248 | -.017 | -1.680 | .093 | -.903 | .070 |
|  | angina | -1.411 | .377 | -.039 | -3.746 | .000 | -2.150 | -.673 |
|  | stroke | -2.238 | .431 | -.052 | -5.195 | .000 | -3.082 | -1.393 |
|  | MI | -.464 | .320 | -.015 | -1.450 | .147 | -1.090 | .163 |
|  | cancer | -.079 | .216 | -.004 | -.367 | .714 | -.503 | .345 |
| a. Dependent Variable: MCS | | | | | | | | |

**Table S3** Likert scores of the frequency with which men had thought about their AAA in the preceding 4 weeks

| **Time Since Initial Screening** | **Likert Score (Mean ±SD)** | **p-value** |
| --- | --- | --- |
| Group 1 (0-12 months) | 2.10 ±1.0 | (reference) |
| Group 2 (13-24 months) | 1.85 ±0.97 | 0.025 |
| Group 3 (25-36 months) | 1.83 ±1.83 | 0.040 |
| Group 4 (37+ months) | 1.75 ±0.87 | 0.005 |

p values calculated using Group 1 data as control

**Table S4** Likert scores of the frequency with which men had thought about their AAA growth in the preceding 4 weeks

| **Time Since Initial Screening** | **Likert Score (Mean ±SD)** | **p-value** |
| --- | --- | --- |
| Group 1 (0-12 months) | 2.16 ±1.1 | (reference) |
| Group 2 (13-24 months) | 1.91 ±1.0 | 0.063 |
| Group 3 (25-36 months) | 1.78 ±0.96 | 0.004 |
| Group 4 (37+ months) | 1.79 ±0.99 | 0.006 |

p values calculated using Group 1 data as control group


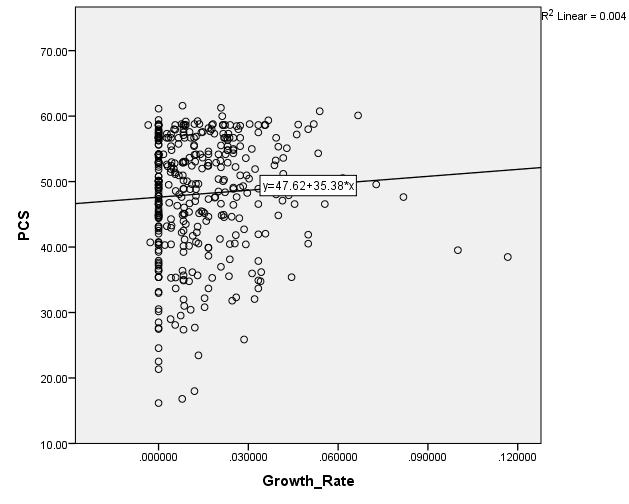


**Fig. S1** Linear regression for aneurysm growth rate and Physical Component Summary score


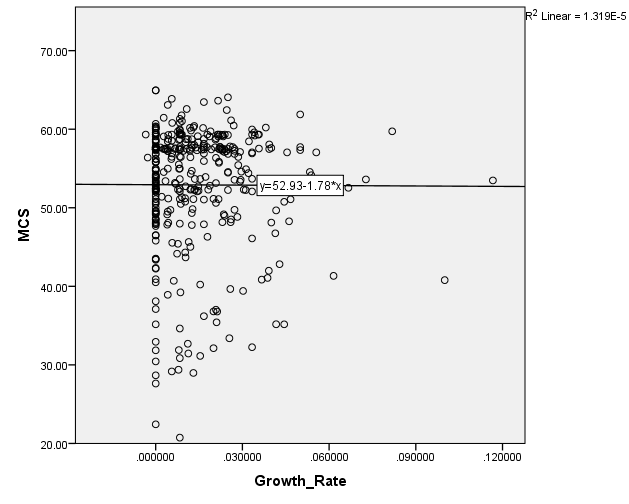


**Fig. S2** Linear regression for aneurysm growth rate and Mental Component Summary score
